# Comparison of the clinical efficacy of bone grafting and bone grafting combined with guided tissue regeneration in periodontal regenerative therapy: a meta-analysis

**DOI:** 10.2340/aos.v83.40255

**Published:** 2024-04-22

**Authors:** Fengqi Zhang, Guolin Liu

**Affiliations:** Department of Stomatology, Liangxiang Hospital of Beijing Fangshan District, Beijing, China

**Keywords:** Bone grafting, bone grafting combined with guided tissue regeneration, periodontal regenerative, meta-analysis

## Abstract

**Objective:**

This study aims to compare the clinical efficacy of simple bone grafting and bone grafting combined with guided tissue regeneration (GTR) in periodontal regenerative therapy.

**Methods:**

The authors systematically searched PubMed, the Web of Science, The National Library of Medicine, the China National Knowledge Infrastructure database and the Wanfang database and collected randomized controlled trials relating to bone graft co-guided tissue regeneration. The retrieval was conducted between January 1990 and December 2022. This study included relevant literature about the clinical efficacy of bone grafting combined with GTR according to the population, intervention, control and outcomes principle and excluded studies using other materials in addition to bone graft and membrane materials. After independently screening the literature, extracting the data and evaluating the risk of bias in the included studies, data analysis was performed using RevMan 5.3 software.

**Results:**

Eighteen studies met the inclusion criteria, and, after further evaluation, a total of 327 teeth that were featured in 15 articles were finally included for meta-analysis. The meta-analysis showed that there was no significant statistical difference in clinical attachment level, probing depth and bone gain between the test group (bone grafting with GTR) and the control group (bone grafting only) at 6 months after the operation (*p* > 0.05). In terms of gingival recession (GR), the use of non-resorbable membranes produced more recession in the test group compared with the control group (*p* < 0.05), whereas the use of resorbable membranes produced less recession (*p* < 0.05).

**Conclusion:**

Both simple bone grafting and bone grafting combined with membrane materials have good clinical efficacy in periodontal regenerative therapy, and no significant difference in clinical efficacy is indicated between the two, with the exception of GR.

## Introduction

Periodontitis is a type of inflammatory disease that occurs in periodontal supporting tissues and is characterized by attachment loss, periodontal pocket formation and alveolar bone resorption. Epidemiological studies have identified that dental caries and periodontitis are the most common oral diseases and major causes of tooth loss; the prevalence of periodontitis is high, with approximately 11.2% of people worldwide suffering from severe periodontal disease. Periodontitis is reported [1] to have affected more than half of the adult population in China, Europe and the United States. Consequently, periodontitis has emerged as a prevalent and pressing public health issue, and its effective treatment and avoidance are increasingly important.

Patients with severe periodontitis may require regenerative therapy to repair supporting tissue around the teeth to achieve periodontal regeneration after non-surgical periodontal therapy. In recent decades, extensive and in-depth studies [2] have been conducted involving four factors that are essential for periodontal regeneration: stem cells, blood supply, scaffold materials and growth. Guided tissue regeneration (GTR) is a technique that involves applying membranous material on gingival connective and epithelial tissues above the defect, thereby blocking the ingrowth of the tissues into periodontal defects.

Bone grafting is a technique in which various natural bone or synthetic materials are implanted to maintain the defect space and induce osteogenesis. A systematic review [3] explored the effectiveness of growth factors in periodontal regeneration, as well as studies [4] applying bone replacement transplantation for the treatment of periodontal bone defects. Other studies [5] have demonstrated the clinical efficacy of various materials and techniques, used alone or in combination. Researchers have also investigated the effectiveness of simultaneous bone grafting and bone grafting combined with GTR for periodontal regeneration in comparative studies. Given the unclear differences in clinical efficacy, we systematically searched relevant studies in this paper and conducted a meta-analysis and systematic evaluation to compare the clinical efficacy of bone grafting alone and bone grafting combined with GTR.

## Materials and methods

### Literature inclusion and exclusion criteria

This study followed the population, intervention, control and outcomes principles and established the following inclusion criteria: population – patients with moderate to severe periodontitis and periodontal bone defects who required periodontal regenerative therapy, confirmed by clinical and imaging examination; intervention – the test group used bone graft material implanted with membrane material; comparison – the control group used only bone graft material; outcome – measurements were taken of clinical attachment level (CAL), probing depth (PD), gingival recession (GR) and vertical bone gain (VBF). The study was designed as a randomized controlled trial (RCT) with a follow-up time of ≥3 months.

The exclusion criteria were as follows: the literature was non-Chinese and non-English; the study used other influences (e.g., growth factors, cell therapy) in addition to bone graft materials and membrane materials; the data could not be extracted from the source; the experiment was on animals; the study was categorized as high risk following a bias assessment.

### Literature search strategy

Following the Preferred Reporting Items for Systematic Reviews and Meta-Analyses instruction manual, three English databases (PubMed, Web of Science and The National Library of Medicine) and two Chinese databases (the China National Knowledge Infrastructure [CNKI] and the Wanfang databases) were systematically searched. The search was conducted between 1 January 1990 and 31 December 2022 and was performed using a combination of subject headings and free words. English search terms included ‘guided tissue regeneration’, ‘GTR’, ‘membrane barrier’, ‘Bio-Gide’, ‘bone transplantation’, ‘bone substitutes’, ‘bone regeneration’, ‘bone graft’, ‘osseous graft’, ‘synthetic graft’, ‘hydroxyapatites’, ‘calcium phosphate’, ‘beta-tricalcium phosphate’, ‘bioactive ceramic graft’, ‘Bio-Oss’, ‘periodontitis’, ‘periodontal bone defect’, ‘periodontal regeneration’, ‘intrabony defect’, ‘infrabony defect’, ‘furcation defect’, ‘periodontal osseous defect’ and ‘furcation involvement’. Chinese search terms included ‘bone grafting’, ‘bone graft’, ‘guided tissue regeneration’, ‘GTR’, ‘barrier membrane’, ‘periodontitis’, ‘bone defect’, ‘intraosseous pocket’ and ‘furcation lesions’. The corpus of included literature was established by employing the specified search terms as keywords and employing the logical operator ‘OR’ for the search. Then – taking the CNKI database as an example – the retrieval strategy was as follows: (Topic: # bone transplantation) AND (Topic: # guided tissue regeneration) AND (Topic: # periodontitis).

In addition to computer retrieval, a manual retrieval of library-related journal articles was conducted and the references were traced in the relevant literature to ensure that the retrieval results were comprehensive and effective.

### Literature screening and data extraction

Two researchers independently screened the literature by first performing preliminary screening through titles and abstracts and then reading the full text according to the inclusion and exclusion criteria for secondary screening. When inconsistent opinions were encountered, the views of a third researcher were solicited and discussed to reach a unified opinion. After the literature screening, data extraction was performed independently by the two researchers and included the following data sets: (1) the basic characteristics of the included studies (i.e., the first author, study type, publication year, publication country, sample size and interventions) and (2) the outcome measures (i.e., CAL, PD, GR and VBF).

### Risk of bias assessment

All included studies were assessed independently by the two researchers for risk of bias. This was carried out using the risk bias assessment tool in the Cochrane Handbook for Systematic Reviews version 5.2.0.

### Statistical analysis methods

A meta-analysis was conducted utilizing RevMan 5.3 software. The effect measure of choice was the standard mean deviation (MD), and each effect size was represented as a point estimate accompanied by a 95% confidence interval (CI). A heterogeneity test was performed to determine heterogeneity according to an *I*^2^ test (*I*^2^ < 50%) or *p* > 0.1. The included literature studies were considered homogeneous and analyzed using the fixed effect model (Mantel–Haenszel); if *I*^2^ > 50% or *p* ≤ 0.1, the included studies were considered to have a degree of heterogeneity and were analyzed using the random effects model (Der Simonian–Laird). In the event of significant heterogeneity, subgroup or sensitivity analyses were conducted to elucidate its origins. The statistical significance threshold for the meta-analysis was established at *α* = 0.05, with a *p*-value < 0.05 deemed as indicative of statistical significance.

## Results

### Literature search results

**Figure 1:**
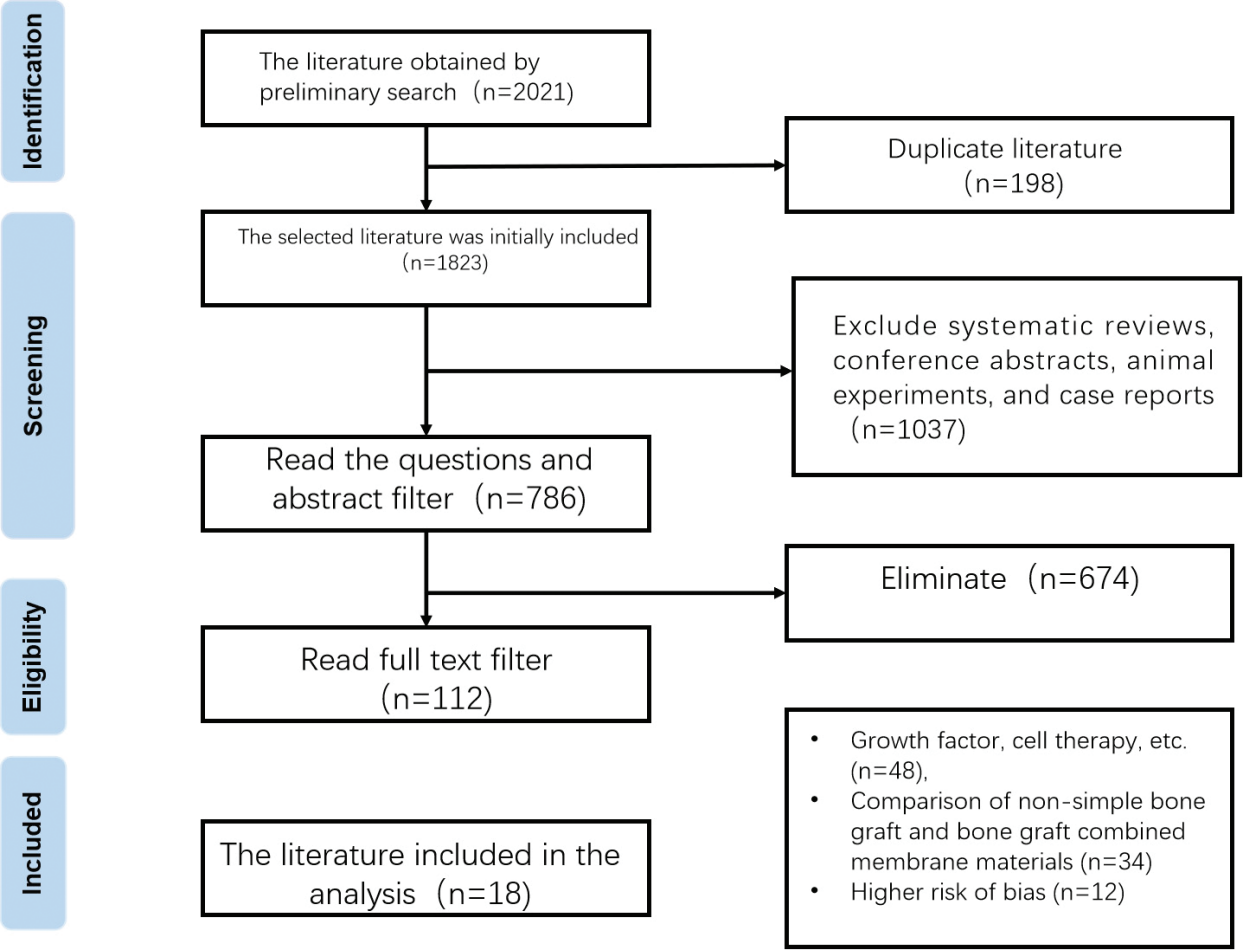
Literature screening and flow chart.

A total of 2,021 relevant literature studies were retrieved by the current search process. After systematic screening, 18 studies [6–23] that met the criteria were finally included for meta-analysis and systematic review. A flow chart of the literature retrieval and screening process is shown in Figure 1.

### Basic characteristics of included studies

The basic characteristics of the included studies are presented in Table 1. Among these, 10 were conducted in India, four in the United States, two in Turkey and the remaining two were conducted in South Korea and China. One study conducted in Turkey (1997) and one in the United States (1993) used non-resorbable membranes [8, 11], and the remaining 16 used resorbable membranes.

**Table 1:** Basic characteristics of the included studies.

Included in the literature	The year of publication	The publication of the country	The type of research	Sample capacity		Intervention study		Follow-up/month	Outcome indicators
				Test team	Control group	Test team	Control group		
Chung et al. ^4^	2014	Korea	Parallel control	10	10	BM+NC	BM	3	1, 2, 3, 4
Lyons et al. ^14^	2008	America	Parallel control	9	11	DFDBA+PLA	DFDBA	9	1, 2, 3, 4
Nygaard-Østby et al. ^19^	2008	America	Parallel control	19	20	AB+PLA	AB	9	1, 2, 3, 4
Tsao et al. ^30^	2006	America	Parallel control	9	9	MBA+CM	MBA	6	1, 2, 4
Srivastava et al. ^27^	2015	India	Parallel control	16	14	Grabio Glascera+PerioCol ™	GrabioGlascera ™	6	1, 2, 4
Agarwal et al. ^1^	2012	India	Parallel control	8	8	DFDBA+PLA	DFDBA	6	1, 2, 4
Mehrotra et al. ^15^	2019	India	Parallel control	5	5	OSTOFORM^TM^+ BioMesh^TM^	OSTOFORM^TM^	6	1, 2, 3
Kini et al. ^11^	2016	India	Parallel control	8	8	CAMCERAM+ Freeflow^TM^	CAMCERAM	6	1, 2, 3
Kilic et al. ^10^	1997	Turkey	Parallel control	10	10	HAC +ePTFE	HAC	6	1, 2, 3, 4
Guillemin et al. ^6^	1993	America	Divide the design	15	15	DFDBA+ePTFE	DFDBA	6	1, 2, 3, 4
Taheri et al. ^29^	2009	India	Divide + parallel	8	10	Bio-Oss+Bio-Gide	Bio-Oss	6	1, 2, 3, 4
Kumar et al. ^12^	2015	India	Divide the design	27	27	Hydroxyapatite+AM	Hydroxyapatite	6	1, 2, 4
Sali et al. ^23^	2016	India	Divide the design	10	10	DFDBA+AM	DFDBA	12	1, 2, 3, 4
Keles et al. ^7^	2010	Turkey	Divide the design	12	12	ACB+PLA	ACB	6	1, 2, 3, 4
Pajnigara et al. ^20^	2017	India	Divide the design	20	20	DFDBA+AM	DFDBA	6	1, 2, 3, 4
Reddy et al. ^21^	2006	India	Divide the design	10	10	Bio-Oss+Bio-Gide	Bio-Oss	6	1, 2, 3, 4
Khashu et al. ^8^	2012	India	Divide the design	6	6	ABM PepGen P-15+Atrisorb	ABM PepGen P-15	9	1, 2, 3, 4
Zhang et al. ^32^	2020	China	Divide the design	21	21	Bio-Oss+Bio-Gide	Bio-Oss	6	1, 2, 3

Note: 1 = PD; 2 = CAL; 3 = GR; 4 = VBF.

BM: bovine bone mineral; NC: nonchemical cross-linking collagen membrane; DFDBA: demineralised freeze-dried bone allograft; PLA: polylactic acid; AB: autogenous bone; MBA: mineralized human cancellous bone allograft; CM: collagen membrane; HAC: hydroxyapatite-collagen alloplastic; ePTFE: expanded polytetrafluoroethylene; AM: amniotic membrane; ACB: autogenous cortical bone; ABM: autogenous cortical bone; OSTOFORMTM: hydroxyapatite with collagen fibres; BioMeshTM: polyglycolide and polylactide copolymer membrane; PerioCol ™: a bioresorbable membrane; Grabio Glascera : a kind of bone graft; CAMCERAM: Biphasic calcium phosphate alloplast; FreeflowTM: synthetic bioabsorbable GTR barrier; Bio-Oss: Bovine xenograft plus 10% collagen; Bio-Gide: porcine bioresorbable collagen barrier; Atrisorb: synthetic bioresorbable barrier membrane.

### Evaluation of the risk of bias of included studies

Results based on the particularity of clinical treatment and the blinding of participants and implementers could not be achieved, and the blinded design of the present study was considered to reflect a low risk of bias when all outcome data in the study were measured by a third-party tester. The assessment results are presented in Table 2, with 13 studies reflecting a low risk of bias and five indicating a moderate risk of bias.

**Table 2:** Risk of bias for the included studies.

Included in the literature	Stochastic method	Blind method	Allocation concealment	The completeness of the results	Selective report	Other	Bear fruit
Chung et al. ^4^	computer	proper	proper	No lost visit	proper	proper	low
Lyons et al. ^14^	computer	proper	proper	Loss to follow-up (1 / 30)	proper	proper	low
Nygaard-Østby et al. ^19^	computer	proper	NK	Lost to follow-up (1 / 40)	proper	proper	low
Tsao et al. ^30^	draw lots	proper	NK	Loss to follow-up (3 / 30)	proper	proper	low
Srivastava et al. ^27^	toss a coin	NK	NK	No lost visit	proper	NK	centre
Agarwal et al. ^1^	NK	NK	proper	No lost visit	proper	proper	centre
Mehrotra et al. ^15^	toss a coin	proper	NK	No lost visit	proper	proper	low
Kini et al.^11^	computer	proper	NK	No lost visit	proper	proper	low
Kilic et al. ^10^	NK	proper	proper	No lost visit	NK	proper	centre
Guillemin et al. ^6^	NK	NK	proper	No lost visit	proper	NK	centre
Taheri et al. ^29^	Grenical sieve	proper	NK	No lost visit	proper	proper	low
Kumar et al. ^12^	computer	proper	proper	Loss to follow-up (3 / 30)	proper	proper	low
Sali et al. ^23^	computer	proper	proper	No lost visit	proper	proper	low
Keles et al. ^7^	toss a coin	proper	proper	No lost visit	proper	proper	low
Pajnigara et al. ^20^	toss a coin	proper	NK	No lost visit	proper	NK	low
Reddy et al. ^21^	toss a coin	proper	proper	No lost visit	proper	proper	low
Khashu et al. ^8^	NK	proper	proper	No lost visit	NK	proper	centre
Zhang et al. ^23^	NK	proper	proper	No lost visit	proper	proper	low

### Meta-analysis results

One Korean and one Indian study (both published in 2016) [7, 19] could not be included in the meta-analysis due to unmatched follow-up times, and an Indian study [13] published in 2015 could not obtain the standard deviation of its 6-month postoperative changes; accordingly, only qualitative analysis was conducted for these three studies. A total of 327 teeth from 15 RCTs [6, 8–12, 14–18, 20–23] were finally included in the meta-analysis. According to the follow-up times, the studies were divided into 6- and 9-month post-surgery groups, and the 6-month group was analyzed according to the different materials used. Their CAL, PD, GR and VBF were analyzed, and the results are shown below.

#### Six months after surgery

Lyons et al. [14] and Mehrotra et al. [15] evaluated the outcome measures CAL, PD, GR and VBF in the 6-month postoperative group, and the results are as follows:

1) CAL: MD = 0.58 with a 95% CI (−0.19, 1.36) in the non-absorbable membrane subgroup [14, 15], indicating there was no significant difference between the test and control groups (*p* = 0.14), and there was no heterogeneity within the group (*I*^2^ = 0%, *p* = 0.40). The MD of the absorbable membrane subgroup [9, 10, 12, 13, 15, 17, 18, 20–23] was 0.65 with a 95% CI (−0.13, 1.64), indicating there was no significant difference between the test and control groups (*p* = 0.56), and there was heterogeneity within the group (*I*^2^ = 85%, *p* < 0.001). There was no significant heterogeneity between the two subgroups (*I*^2^ = 0%, *p* = 0.25).

2) PD: MD = 0.88, with a 95% CI (−1.22, 2.98) in the non-absorbable membrane subgroup [14, 15], indicating there was no significant difference between the test and control groups (*p* = 0.41), and there was heterogeneity within the group (*I*^2^ = 85%, *p* = 0.009). The MD of the absorbable membrane subgroup [9, 10, 12, 13, 15, 17, 18, 20–23] was 0.52, with a 95% CI (−0.03, 1.45), indicating there was no significant difference between the test and control groups (*p* = 0.07), and there was heterogeneity within the group (*I*^2^ = 83%, *p* < 0.001). No significant between-group heterogeneity existed among the two subgroups (*I*^2^ = 3%, *p* = 0.45).

3) GR: In the non-absorbable membrane subgroup [8, 11], there was 0.55 mm more GR in the test group than in the control group, and the difference was statistically significant (*p* = 0.02), with no within-group heterogeneity (*I*^2^ = 0%, *p* = 0.83). In the absorbable membrane subgroup [9, 10, 12, 15, 17, 18, 21, 23], the GR of the test group was 0.32 mm less than that of the control group, and the difference was statistically significant (*p* = 0.01), with no within-group heterogeneity (*I*^2^ = 0%, *p* = 0.57). Significant between-group heterogeneity existed among the two subgroups (*I*^2^ = 95%, *p* = 0.001).

4) VBF: In the non-absorbable membrane subgroup [8, 11], the VBF of the test group was 0.23 mm more compared with the control group, and the difference was statistically significant (*p* = 0.02), with heterogeneity within the group (*I*^2^ = 67%, *p* = 0.005). In the absorbable membrane subgroup [6, 9, 13, 17, 18, 20–22], the VBF of the test group was 0.42 mm more compared with the control group, and the difference was statistically significant (*p* = 0.01), with heterogeneity within the group (*I*^2^ = 44%, *p* = 0.001). There was significant heterogeneity between the two subgroups (*I*^2^ = 67%, *p* = 0.01).

#### Nine months after surgery

Lyons et al. [14], Nygaard et al. [16] and Khashu et al. [10] evaluated the outcome measures CAL, PD, GR and VBF in the 9 months postoperative group, and the results are as follows:

1. CAL [10, 14, 16]: The difference between the test and control groups was not statistically significant (*p* = 0.38), and there was no heterogeneity within the group (*I*^2^ = 0%, *p* = 0.83).
2. PD [10, 14, 16]: The difference between the test and control groups was not statistically significant (*p* = 0.90); there was no heterogeneity within the group (*I*^2^ = 0%, *p* = 0.41).
3. GR [10, 14, 16]: This result was 0.58 mm more in the test group compared with the control group, and the difference was statistically significant (*p* = 0.03), with heterogeneity within the group (*I*^2^ = 27%, *p* = 0.26).
4. VBF [10, 14, 16]: The difference between the test and control groups was not statistically significant (*p* = 0.32), and there was heterogeneity within the group (*I*^2^ = 7%, *p* = 0.30).

#### Publication bias analysis

**Figure 2:**
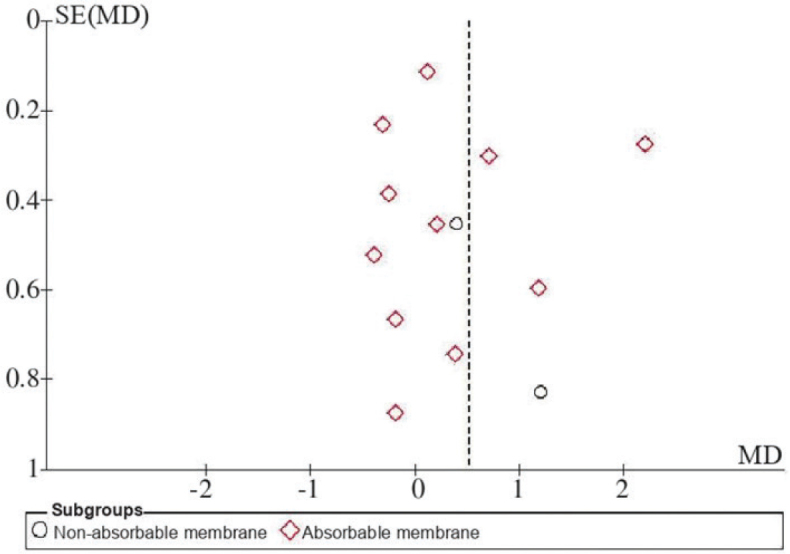
Funnel plot of CAL 6 months post-op.

Publication bias analysis was performed for the included studies, with MD as the abscissa and the standard error of MD as the ordinate. The ‘funnel plot’ of CAL indicators at 6 months after surgery is shown in Figure 2 and shows that 1 of 13 studies [17] indicated significant deviation, and the remaining studies reflected no significant symmetry, suggesting that there may have been some publication bias.

#### Sensitivity analysis

Sensitivity analysis revealed that heterogeneity within groups was significantly reduced when articles including CAL indicators [17], PD indicators [15], VBF indicators [22] and articles in literature [17] were excluded. The meta-analysis showed that the heterogeneity (*I*^2^) value between GR subgroups was as high as 95%, and the membrane materials used in the studies that included GR indicators could be divided into four categories: non-absorbable expanded polytetrafluoroethylene (ePTFE) membrane [8, 11]; absorbable polylactic acid membrane [9]; amniotic membrane (AM) [17]; Bio-Gide^®^ membrane [18, 21]. The results after removing one type of membrane material are detailed in Table 3 and show that between-group heterogeneity was significantly reduced only when the non-resorbable membrane was removed and remained high when the other resorbable membranes were removed. The results of the sensitivity analysis suggest that the use of non-absorbable and absorbable membranes may produce different GR conditions.

**Table 3:** Results of the sensitivity analysis.

Outcome index	Included in the study	Excluding the study	Results of the heterogeneity test		Meta analysis of the results		Effect model
			*I*^2^ (Intra-group%)	*I*^2^ (interblock%)	MD (95% CI)	*p*	
CAL	[30, 27, 1, 15, 11, 29, 12, 7, 21, 8, 32]	[20]	46	-	0.34 (−0.09, 1.23)	0.67	fixed
PD	[30, 27, 1, 11, 29, 12, 7, 21, 8, 32]	[15, 20]	53	-	0.48 (−0.12, 1.38)	0.08	fixed
VBF	[27, 1, 29, 12, 7, 20, 21]	[30]	17	-	0.53 (0.14, 2.05)	<0.001	fixed
GR	[15, 11, 29, 7, 20, 21, 8, 32]	[10, 6]	0	18	−0.32 (−0.46, −0.05)	0.01	stochastic
GR	[15, 11, 29, 20, 21, 8, 32]	[7]	25	67	0.19 (−0.02, 0.52)	0.78	stochastic
GR	[15, 11, 29, 7, 21, 8, 32]	[20]	34	52	0.06 (−0.08, 0.27)	0.56	stochastic
GR	[15, 11, 7, 20, 8, 32]	[29, 21]	67	84	0.04 (−0.12, 0.16)	0.09	stochastic

CAL: clinical attachment level; PD: probing depth; GR: gingival recession; VBG: vertical bone gain; MD: mean deviation; CI: confidence interval.

At 9 months after surgery, no significant heterogeneity was observed in any of the four clinical indicators, and the *I*^2^ values were <50%. The reason for this may have been that the number of studies was too small and the membranes used in all three studies were absorbable.

#### Qualitative analysis

A study conducted by Chung et al. [7] reported changes related to CAL, PD and bone gain 3 months after surgery and showed that the reduction of PD in an nonchemical cross-linking collagen membrane (NC) + xenograft bone mineral (BM), bilayer collagen membrane (BC) + BM and BM groups was (5.10 ± 1.52), (3.60 ± 1.27) and (3.60 ± 2.12) mm, respectively; the increase in CAL was (3.60 ± 2.76), (2.50 ± 1.72) and (2.50 ± 3.41) mm, respectively, and the imaging bone gain was (5.83 ± 3.68), (5.0 2 ± 2.39) and (6.9 5 ± 2.43) mm, respectively. Compared with the baseline, clinical outcomes were significantly improved in all three groups at 3 months after surgery, but there were no significant differences between them. The results were similar at a 12-month follow-up by investigators [19]. The CAL, PD and bone depth in periapical radiograph and cone beam computed tomography images were significantly improved in both the test and control groups, but there was no significant difference between the two groups. In addition, no significant GR was observed in the test and control groups in this study.

Kumar et al. [13] used an AM as a barrier material in the test group, and their results showed significant improvements in PD, CAL and VBF at baseline compared with 12 and 24 weeks after surgery in both treatment groups. When compared between the groups, PD was significantly reduced in the test group at 24 weeks after surgery; CAL was also significantly increased in the test group at both 12 and 24 weeks after surgery, and VBF was significantly increased in the test group compared with the control group at 24 weeks after surgery. These results were slightly different from the results of other studies.

## Discussion

Bone grafting and GTR combined with bone grafting have been widely used in clinical practice and have become primary treatment methods for periodontal diseases and periodontal intraosseous defects. However, it is not clear whether there is a difference in the clinical efficacy of the two procedures. This study compared and analyzed the clinical efficacy of the two procedures via a meta-analysis and systematic review.

Based on the sensitivity analysis results, the heterogeneity of the overall results changed after removing part of the literature. Factors that may contribute to heterogeneity include the type of membrane, surgical technique, the frequency of maintenance therapy and detection methods [24]. The results of this study were confirmed in existing research. For example, Kianye et al. [25] compared the clinical efficacy of AM and collagen membranes and found their effectiveness to be similar; however, the AM could yield less GR. In addition, the heterogeneity of bone augmentation may be related to the measurement methods, projection angles, machines used, imaging quality and measurement differences between examiners. No significant symmetry was observed in the funnel plot of CAL indicators at 6 months after surgery, and one study showed significant deviation, indicating the possible presence of publication bias [17]. This may be because the number of studies included for this indicator was too small and primarily distributed in the middle of the funnel plot, suggesting that the study’s accuracy was moderate and that there was a lack of sufficient large-sample studies. Furthermore, the bone graft and membrane materials used in each study differed, and the heterogeneity generated among studies would also have had some impact on the symmetry of the funnel plot [26].

The results of this study’s meta-analysis showed that among the outcome measures, only the GR was significantly different between the bone graft groups, and the degree of GR was significantly different between the absorbable membrane and non-absorbable membrane subgroups. At 6 months after surgery, the use of a non-resorbable membrane showed a more pronounced GR in the test group, whereas the use of a resorbable membrane showed a lower GR in the test group. The results of this study suggest that the use of membrane material in periodontal regenerative surgery is beneficial for treating GR [27]. However, relevant studies [28] also found that for non-absorbable membranes, the removal of membrane materials requires secondary surgery and the exposure of membrane materials during the healing process, which may lead to additional GR.

In addition, some clinical studies [29, 30] suggested that the use of membrane materials may cause complications, such as the failure of primary healing, dehiscence of the gingival flap, membrane exposure, suppuration and the promotion of bacterial growth. When the ePTFE membrane was placed over the defect for more than 8 weeks, few vessels had developed below the membrane and vascular anastomosis between the periodontal ligament and gingival tissue had not been established. Thus, the placement of non-resorbable membranes may affect the reconstruction of the vascular network on the alveolar ridge; however, similar studies have not been found for resorbable membranes, particularly collagen membranes.

For bone grafting only, some clinical and histological studies [31, 32] have demonstrated that periodontal regeneration could be achieved. Systematic reviews of clinical studies have shown that the morphology of bone defects plays a crucial role in the process of defect healing, and studies found that combined treatment did not produce better outcomes in the treatment of 3-wall intraosseous pockets, second-degree furcation lesions or fenestrated defects. In supraosseous and 2-wall infraosseous pouch defects, the combined treatment showed superior histological results in terms of bone augmentation [33]. The study indicated no significant statistical difference in PD, CAL or GR between the test and control groups from 6–12 months after regeneration. The above study results were essentially consistent with the results of the current meta-analysis.

This systematic review has some limitations. First, not all the studies mentioned whether patients who smoked and/or had a history of systemic diseases had been excluded, which may have had an impact on the outcome. These two types of patients typically show poor responsiveness to periodontal treatment, which can slow the patient’s periodontal recovery and may have an impact on outcome measures, such as PD. Second, some publication bias may have been present in the selected studies; as such, more high-quality, large-sample-size clinical studies are needed. Finally, too few studies with long-term follow-up measures were included, and the stability of the results requires additional confirmation.

In summary, bone grafting combined with GTR displayed significant differences in clinical efficacy, specifically for GR. The findings indicate the potential use of absorbable membrane materials to mitigate GR risk in patients with thin gingival biotypes undergoing periodontal regenerative therapy. Furthermore, larger and more comprehensive long-term studies are needed for pro-angiogenic materials to facilitate loss repair.
